# Paired refinement under the control of *PAIREF*

**DOI:** 10.1107/S2052252520005916

**Published:** 2020-06-10

**Authors:** Martin Malý, Kay Diederichs, Jan Dohnálek, Petr Kolenko

**Affiliations:** a Faculty of Nuclear Sciences and Physical Engineering, Czech Technical University in Prague, Břehová 7, Prague 11519, Czech Republic; b Institute of Biotechnology of the Czech Academy of Sciences, Biocev, Průmyslová 595, Vestec 25250, Czech Republic; c University of Konstanz, Box M647, Konstanz 78457, Germany

**Keywords:** macromolecular crystallography, *PAIREF*, X-ray diffraction, paired refinement, high-resolution limit

## Abstract

Crystallographic resolution is a key characteristic of diffraction data and represents one of the first decisions an experimenter has to make in data evaluation. Conservative approaches to the high-resolution cutoff determination are based on a number of criteria applied to the processed X-ray diffraction data only. However, high-resolution data that are weaker than arbitrary cutoffs can still result in the improvement of electron-density maps and refined structure models. Therefore, the impact of reflections from resolution shells higher than those previously used in conservative structure refinement should be analysed by the paired refinement protocol. For this purpose, a tool called *PAIREF* was developed to provide automation of this protocol. As a new feature, a complete cross-validation procedure has also been implemented. Here, the design, usage and control of the program are described, and its application is demonstrated on six data sets. The results prove that the inclusion of high-resolution data beyond the conventional criteria can lead to more accurate structure models.

## Abstract 2

Application of the *PAIREF* program providing automation of paired refinement is demonstrated on six data sets. The results prove that the inclusion of high-resolution data beyond the conventional criteria can lead to more accurate structure models.

## 1. Introduction   

Crystallographic resolution is understood as the minimum plane spacing given by Bragg’s law for a particular set of X-ray diffraction intensities that are included in the structure analysis (*Online Dictionary of Crystallography*, https://dictionary.iucr.org/Resolution). In contrast, optical resolution is defined as the expected minimum distance between two resolved peaks in the electron-density map (Vaguine *et al.*, 1999 ▸). The resolution of data is limited due to a decrease in the intensity-to-noise ratio of reflections with the resolution. The weakness of the high-resolution data is caused by several factors, including the Lorentz-polarization factor, temperature factor and crystal imperfection. Therefore, the diffraction data are usually cut off at a certain resolution, with the aim of rejecting the data that do not improve the model.

In previous decades, conservative criteria were applied to estimate the resolution of crystallographic data. These criteria were based on a user-defined value of data quality indicators such as the signal-to-noise ratio 〈*I*/σ(*I*)〉, the disagreement residual of multiple observations *R*_merge_, *etc.* (Evans, 2011 ▸)*.* Later, the Pearson correlation coefficient CC_1/2_, quantifying the internal consistency of observations, was added to these criteria (Karplus & Diederichs, 2012 ▸). Inspection of the data deposited in the PDB (Berman *et al.*, 2000 ▸) shows that there is no consensus in the application of these statistics. Moreover, the possibility of improvement of a refined model by employing a different resolution range was often not considered. Nowadays, the application of strict cutoff values on selected data quality indicators has been shown to be an obsolete approach (Diederichs & Karplus, 2013 ▸; Evans & Murshudov, 2013 ▸). Very recently, it became possible to estimate the information gain from each reflection using likelihood-based methods (Read *et al.*, 2020 ▸). Yet this approach does not answer the question of which high-resolution cutoff should be used with current refinement programs.

The ambiguity in the high-resolution-cutoff estimation has been removed with the advent of the ‘paired refinement’ protocol (Karplus & Diederichs, 2012 ▸). Initially, a conservative criterion is applied as usual to the high-resolution data and the phase problem is solved. Usually, the model is then significantly improved by refinement. In the paired refinement protocol, the influence of the previously rejected high-resolution data during the structure refinement is tested. The structure model is refined stepwise against data at higher and higher resolution until no improvement of the model is observed. More specifically, each increase in resolution is checked against the original resolution for its added value, particularly by comparing *R* values of models against the same data. Only those resolution shells that prove beneficial are included in the final data set, against which the structure is refined.

In this paper, we present a new tool – *PAIREF* – which helps to make the decision about the useful resolution of the data set. The program performs paired refinement for validation of the high-resolution data in a fully automatic way. *PAIREF* is not the first utility that implements paired refinement since a similar function is present in *PDB-REDO* (Joosten *et al.*, 2014 ▸). Nevertheless, *PAIREF* provides additional features (*e.g.* complete cross-validation, modification of the structure refinement protocol) and reports that naturally require more extensive input, and allows a user to make a more sophisticated decision.

## 2. Design and implementation   

*PAIREF* is a command-line tool that can be installed as a module into the *CCTBX* toolbox (Grosse-Kunstleve *et al.*, 2002 ▸) on various platforms (GNU/Linux, MS Windows). Currently, it has been developed in Python 2.7 (Hunter, 2007 ▸; Rossum, 1995 ▸) but is ready to move to Python 3. It depends on the following programs of the *CCP*4 software package (Winn *et al.*, 2011 ▸): *REFMAC*5 (Murshudov *et al.*, 2011 ▸), *SFCHECK* (Vaguine *et al.*, 1999 ▸), *MTZDUMP*, *SFTOOLS *and *BAVERAGE*; and on the module *pdbtools* (Adams *et al.*, 2010 ▸) from *CCTBX*. Input parameters can be specified in order to place the protocol under the full control of the user.

A typical command-line example for a *PAIREF* job is cctbx.python -m pairef --XYZIN starting_model_2-4A.pdb --HKLIN data_2A.mtz --HKLIN_UNMERGED data_2A_unmerged.mtz -i 2.4 -r 2.3,2.2,2.1,2.0, which executes refinements of the structure model starting_model_2-4A.pdb (previously refined at 2.4 Å) for a series of cutoffs (stepwise 2.3, 2.2, 2.1 and 2.0 Å). Specification of unmerged data (MTZ, unmerged *Scalepack* or *XDS*/*XSCALE* file types) is only required if comparison of CC_work_ with CC* (see below) should be enabled.

### 2.1. Parameters and algorithm   

**Figure 1:**
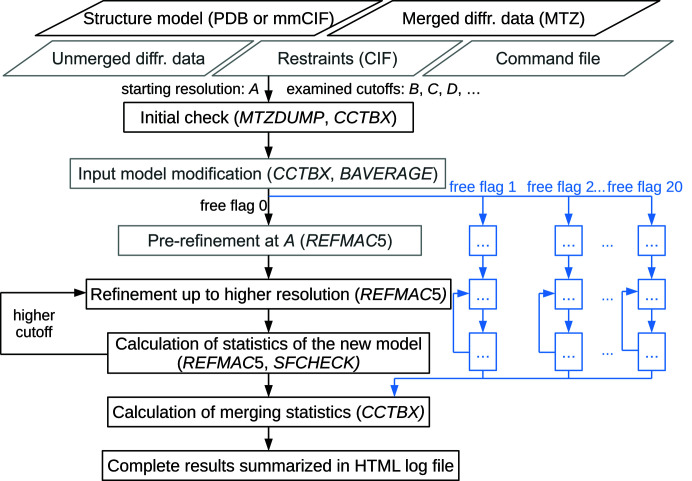
Schematic diagram of the *PAIREF* algorithm. Optional input files and routines are drawn in grey, the complete cross-validation protocol is outlined in blue.

The algorithm implemented in *PAIREF* depends on the amount of data provided by the user. The minimal function of the program requires the following input files: structure model refined at the starting resolution (PDB or mmCIF format) and higher-resolution merged diffraction data in MTZ format which have the same free reflection flags as the data previously used in the refinement (Fig. 1 ▸). Nevertheless, the minimal requirement is not sufficient for deep data analysis including statistics such as CC*, *etc.* The protocol can be further supplemented by the full-resolution unmerged data for calculating merging statistics, by the external restraints in CIF format in the case where non-standard ligands are present and by the command file for *REFMAC*5 (alternatively generated by *PDB-REDO*) for better control of the structure refinement. Moreover, a definition of domains for translation–libration–screw (TLS) refinement can be provided by the user. The program allows the selection of resolution shells (with a default width of 0.05 Å) and optional model modifications before the paired refinement.

Our paired refinement protocol with *REFMAC*5 is an adaptation of the original protocol that has been performed with *phenix.refine* (Karplus & Diederichs, 2012 ▸; Afonine *et al.*, 2012 ▸). Initially, the input files are checked using *MTZDUMP* and *CCTBX* for consistency. The model is then refined against the data up to resolution *B* (higher than *A*), and this model is compared with the original one – both against the data at resolution *A* (see Section 2.2). This step is then repeated from resolution *B* up to resolution *C* (higher than *B*) and reproduced again until the maximum limit is reached. CC_work_ and CC_free_ statistics are calculated using *SFTOOLS* (Karplus & Diederichs, 2012 ▸). Finally, merging statistics are calculated using the *CCTBX* library if unmerged diffraction data were provided.

As an option, *PAIREF* provides a complete cross-validation protocol (Brünger, 1993 ▸; Jiang & Brünger, 1994 ▸) – also referred to as *k*-fold cross-validation (Luebben & Gruene, 2015 ▸) – to investigate the impact of the selection of free reflections. Here, the paired refinement protocol is run in parallel for each selection individually. To remove the bias given by previous refinement with a particular set of free reflections, a number of optional input model modifications prior to refinement have been implemented: the perturbation of the atomic coordinates, the reset of atomic displacement parameters (ADPs) to a particular or average value and the addition of a fixed value to them (achieved by module *pdbtools* from *CCTBX* and *BAVERAGE*). In the final report, both the averaged statistics as well as the individual statistics for each selection are reported. Application of this protocol is demonstrated on a data set from cysteine di­oxy­genase (Section 3.3). The complete cross-validation requires the *CCP*4-style test set description in the input MTZ file, *i.e.* multiple free reflection labels must be present.

The program *PAIREF* does not have any decision-making routines and it remains up to the user to decide on the resolution cutoff based on the comprehensive analysis that was performed. Structure refinement is a multiparametric calculation and the user should be aware of potential problems. For example, nonconvergent refinement may result in misleading statistics and a suboptimal model (Tickle, 2011 ▸). One of the parameters that may potentially play a role is the FFT grid size (Drenth & Jeroen, 2010 ▸).

### 2.2. Program output and interpretation of results   

Paired refinement does not reduce the problem of high-resolution cutoff estimation to a single monitoring statistic. Rather, a comprehensive data analysis is summarized on an HTML page. Here, various plots, tables and links to many intermediate files and log files are presented or easily accessible via hyperlinks.

The first monitoring statistics reported by *PAIREF* are the differences in *R* values between the models refined at adjacent resolutions (both computed at the lower resolution to provide a valid comparison). A decrease in *R*_free_ is expected in shells beneficial to the model quality. However, a constant *R*_free_ and a simultaneous increase in *R*_work_ are usually acceptable as well because these indicate less overfitting of the structure model (Karplus & Diederichs, 2012 ▸). Therefore, the next monitoring statistic is *R*_gap_ (*R*_gap_ = *R*_free_ − *R*_work_) which is calculated at the starting resolution (corresponding to resolution *A* in Section 2.1) for all analyzed shells. This is an implementation of a previously published protocol (Winter *et al.*, 2018 ▸). In the case of the complete cross-validation protocol, *R* values for each set of free reflections and average values are reported. Moreover, the standard deviations of *R* values of structure models refined using different free reflection sets are calculated (Kleywegt & Brünger, 1996 ▸).

However, the overall *R* values are not the only parameters to be taken into account when deciding on the high-resolution cutoff. The analysis is further supplemented by plots of *R*_work_, *R*_free_, CC_work_ and CC_free_ (CC_work_ and CC_free_ are correlation coefficients between experimental and calculated intensities) of the refined structure models at defined resolution. Since a perfect model gives an *R* value of 0.42 against random data (*i.e.* pure noise) – assuming non-tNCS (translational non-crystallographic symmetry) data from a non-twinned crystal (Evans & Murshudov, 2013 ▸) – a higher *R* value in the (current) high-resolution shell indicates either the involvement of high-resolution data without information content (the data are even worse than noise), or poor quality of the model, or the presence of tNCS.

When unmerged data are available, values of CC* are added to the CC_work_ and CC_free_ plots. Comparison of CC values (correlation coefficients) with CC* serves for direct linking of the data and structure model quality (Diederichs & Karplus, 2013 ▸; Karplus & Diederichs, 2012 ▸). CC_work_ or CC_free_ greater than CC* in a high-resolution shell indicates undesirable overfitting of the structure model as the calculated intensities agree with the observed data better than the (usually unavailable) true data. Owing to the independence of CC* on a model, its comparison with CC_work_ is just as informative as comparison with CC_free_. However, the usage of CC_work_ should be preferred since it is based on much more data.

For additional information, *PAIREF* reports the optical resolution as calculated using *SFCHECK* for each resolution cutoff. When all previous procedures are finished and unmerged diffraction data are available, the merging statistics are listed in a table and shown in graphs. Finally, the progress of the refinement procedures is reported to check for convergence *etc*.

### 2.3. Distribution and documentation   

Full documentation of *PAIREF* is available online at https://pairef.fjfi.cvut.cz and the program is distributed at https://pypi.org/project/pairef/.

## 3. Examples   

The functionality and versatility of *PAIREF* have been thoroughly tested on a number of cases. Here, we selected six structures and data sets to demonstrate the broad application potential of the tool: simulated data for lysozyme from *Gallus gallus* (SIM) (Holton *et al.*, 2014 ▸), and measured data for thermolysin from *Bacillus thermoproteolyticus* (TL) (Winter *et al.*, 2018 ▸), a cysteine-bound complex of cysteine di­oxy­genase from *Rattus norvegicus* (CDO) (Karplus & Diederichs, 2012 ▸), endothia­pepsin from *Cryphonectria parasitica* in complex with fragment B53 (EP) (Huschmann *et al.*, 2016 ▸), interferon gamma from *Paralichthys olivaceus* (POLI) (Zahradník *et al.*, 2018 ▸) and bilirubin oxidase from *Myrothecium verrucaria* (BO) (Koval’ *et al.*, 2019 ▸). All the results are available from http://doi.org/10.5281/zenodo.3687267.

A comprehensive summary of crystallographic data as well as the refinement statistics are shown in Tables 1 ▸ and 2 ▸. To be consistent with the previous results, the free reflection flags from the original data were preserved except for TL, because of inaccessibility.

### 3.1. Simulated data set of lysozyme   

The ability to generate artificial X-ray diffraction patterns based on a well defined ‘true’ structure offers the possibility of monitoring the progress of paired refinement, especially the convergence of the refined models towards the ‘true’ structure.

We generated one hundred diffraction images using a modified structure of lysozyme (data set SIM). At first, all alternative conformations were removed from the structure with the PDB entry 1h87 (originally determined at 1.72 Å resolution) (Girard *et al.*, 2002 ▸). The data collection was simulated using *MLFSOM* (Holton *et al.*, 2014 ▸) with a crystal-to-detector distance of 150 mm. *MLFSOM* also simulated global radiation damage for a beam of 8.4 × 10^10^ photons s^−1^ and 100 µm diameter, exposure of 0.1 s and a crystal size of 77.8 µm. Afterwards, the diffraction data set was processed using *DIALS*/*AIMLESS* (Evans & Murshudov, 2013 ▸; Winter *et al.*, 2018 ▸) or *XDS*/*XSCALE* (Kabsch, 2010 ▸) up to a resolution of 1.20 Å, although the CC_1/2_ values become not significantly different from zero (at the 1:1000 level) at 1.35 Å resolution.

The input model for paired refinement was generated from the structure used for the generation of the diffraction images by perturbation of atomic coordinates by an average of 0.25 Å; the ADPs were set to their mean value (15 Å^2^). In the final preparation step, several cycles of restrained refinement at the starting resolution (1.72 Å) against the processed simulated data were performed. In the next step, we performed the paired refinement protocol using *PAIREF*.

**Figure 2:**
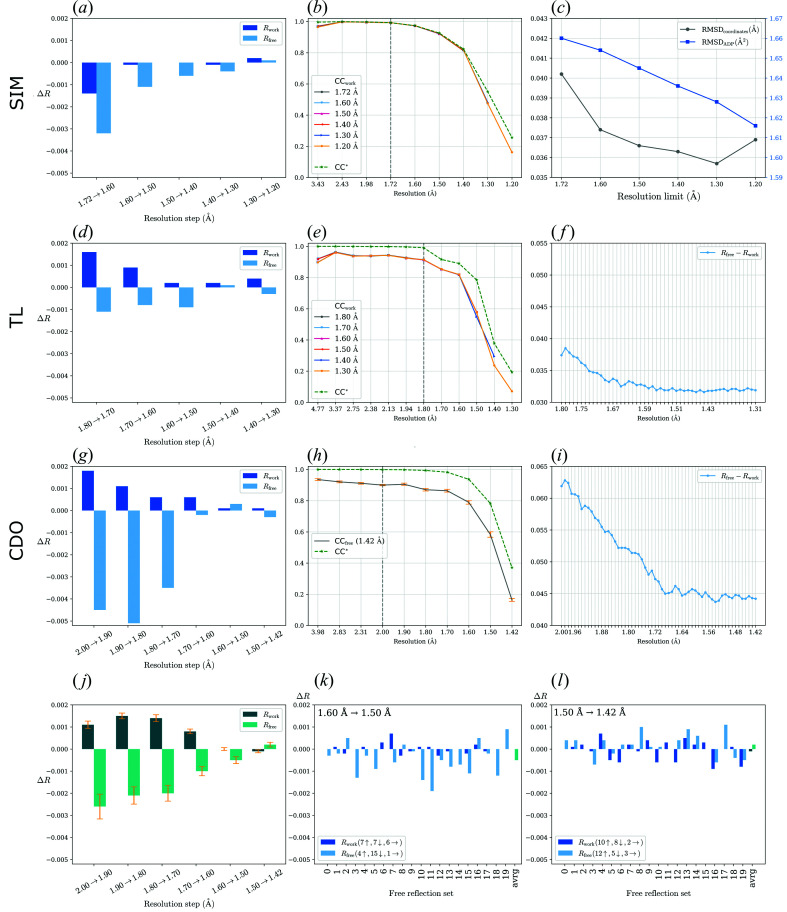
Results from paired refinement for SIM (*a*)–(*c*), TL (*d*)–(*f*) and CDO (*g*)–(*l*). Note for bar charts showing the differences in the overall *R* values: for each incremental step of resolution for *X*→*Y*, the *R* values were calculated at resolution *X*. SIM: (*a*) differences in the overall *R* values; resolution shells with a width of 0.10 Å were added stepwise. *R*_free_ decreases up to 1.30 Å. (*b*) Comparison of CC* and CC_work_ of refined models. (*c*) Both RMSDs of the coordinates and the ADPs (RMSD_coordinates_ and RMSD_ADP_) have a decreasing trend up to 1.3 Å resolution. TL: (*d*) differences in the overall *R* values; resolution shells with a width of 0.10 Å were added stepwise. (*e*) Comparison of CC* and CC_work_ of the refined models. (*f*) *R*_gap_ calculated using data up to 1.80 Å depending on the high-resolution cutoff; resolution shells with a width of 0.01 Å were added stepwise (a different *PAIREF* run, see the supporting information). CDO: (*g*) differences in the overall *R* values; resolution shells with a width of 0.10 Å were added stepwise. (*h*) Comparison of CC* and CC_free_ of the model refined at 1.42 Å, averaged over all of the 20 free sets. The standard error of the mean is shown in orange. (*i*) *R*_gap_ calculated using data up to 2.00 Å depending on the high-resolution cutoff; resolution shells with a width of 0.01 Å were added stepwise (a different *PAIREF* run, see the supporting information). (*j*) Differences in the overall *R* values averaged over all 20 free sets. The standard error of the mean is shown in orange. (*k*) and (*l*) Differences in the overall *R* values relating to all 20 free sets, refinements at 1.50 and 1.42 Å, respectively. The numbers with arrows in the legends indicate how many rises and falls were observed while using individual free reflection sets.

Structure models refined against the simulated data set have considerably lower *R* values when compared with the other structures (based on real experimental data) mentioned later (*R*_free_= 0.071 for SIM versus *R*_free_= 0.195 for TL, both at 1.72 Å). This effect, caused by the simulated character of the data, was also observed in the original work by Holton *et al.* (2014 ▸). However, the trends of nearly all indicators of data quality are similar to those of the real cases [see Fig. 2 ▸(*a*)]. Based on the plot of stepwise differences in overall *R* values, we decided to estimate the high-resolution limit as 1.3 Å because the *R* values increase for resolution shells beyond that limit.

We monitored the root-mean-square deviation (RMSD) values (DeLano Scientific, 2017 ▸) calculated on all 1217 atoms of the simulated structure with respect to the original structure model [Fig. 2 ▸(*c*)]. A systematic decrease was observed for the atomic coordinates when reflections from an additional high-resolution shell were added to the refinement up to 1.3 Å resolution. This is in agreement with the high-resolution cutoff based on the differences in overall *R*-values behaviour only. In general, the RMSD of ADP values calculated for all the atoms (see equation given in the supporting information) follow a similar but not identical trend. Moreover, they continue to decrease and converge to the ‘true’ value even for the highest resolution shell which was later omitted from the data based on the other data quality indicators. As a result of our calculations, we suggest here application of a high-resolution cutoff at 1.3 Å when using our combination of programs and following our refinement protocol. Similar results were also obtained using *XDS*/*XSCALE* for data processing.

### 3.2. Thermolysin   

Successful application of paired refinement was previously demonstrated on the crystal structure of thermolysin (TL) from *B. thermoproteolyticus* (Winter *et al.*, 2018 ▸). In the original protocol, the structure was modified (perturbation of atomic positions) and refined at a defined high-resolution limit in the range from 1.80 to 1.50 Å. Model improvement was monitored on *R*_gap_ only, which decreased until 1.56 Å resolution. A further increase in the resolution did not cause a substantial change of *R*_gap_.

To reproduce most of the original procedures by Winter *et al.*, the diffraction data were processed with *xia2* (Winter, 2010 ▸) using *DIALS*/*AIMLESS* software. The structure of thermolysin (PDB entry 3n21; Behnen *et al.*, 2012 ▸) was used as a starting model. The atomic coordinates were perturbed and all ADPs were generally set to their average value of 22 Å^2^ with *phenix.pdbtools* (Adams *et al.*, 2010 ▸). A total of 30 cycles of restrained refinement were performed with *REFMAC*5 at a resolution of 1.80 Å. After that, ligands (peptide in the active site, three molecules of DMSO) and solvent were built in *Coot* (Emsley *et al.*, 2010 ▸), refined with *REFMAC*5 and finally used in *PAIREF* to analyse the high-resolution cutoff.

We performed two *PAIREF* runs that added stepwise high-resolution shells with a width of 0.10 and 0.01 Å. *R*_free_ has a decreasing trend up to 1.50 Å for the first run [Fig. 2 ▸(*d*)], which suggests that the data should be cut at this resolution. Moreover, the plot of *R*_gap_ [Fig. 2 ▸(*f*)] from the second run further confirms a good agreement between the previously published results and our calculations.

### 3.3. Cysteine di­oxy­genase   

The cysteine-bound complex of cysteine di­oxy­genase from *R. norvegicus* (CDO) (Simmons *et al.*, 2008 ▸) was the first macromolecular crystal structure on which the paired refinement protocol was demonstrated (Karplus & Diederichs, 2012 ▸). Although the conservative criterion for *R*_meas_ suggests setting the high-resolution diffraction limit to 1.80 Å, having 〈*I*/σ(*I*)〉 higher than 2 suggests setting the limit to 1.60 Å, but paired refinement proved that data are useful up to 1.42 Å. All refinement was previously performed using *phenix.refine* (Afonine *et al.*, 2012 ▸).

Here, we tried to reproduce the previous results in *PAIREF* which uses *REFMAC*5 as a structure refinement program. We have reprocessed the original images with *XDS*. The input structure model was prepared according to the following protocol: the protein atomic positions of the unliganded CDO structure (PDB entry 2b5h; Simmons *et al.*, 2006 ▸) were perturbed by an average of 0.25 Å with *phenix.pdbtools*; the ligand (cysteine persulfenate) was built manually with *Coot*. Subsequently, the model was refined with *REFMAC*5 at 2.00 Å resolution, solvent was added automatically using *ARP*/*wARP* (Lamzin & Wilson, 1993 ▸) followed by a manual check of the ligand and solvent and restrained refinement with *REFMAC*5. This model was later used as the input file for *PAIREF* to analyze the high-resolution shells with a width of 0.10 Å. Unlike the protocol published previously, solvent molecules were not automatically updated during paired refinement.

The differences of overall *R* values [Fig. 2 ▸(*g*)] indicate that the high-resolution diffraction limit may be set to 1.60 Å using our combination of software and free reflection set. However, the selection of free reflections may have an impact on the results and conclusions from paired refinement; therefore, we ran the second procedure of 20-fold cross-validation across all free reflection sets, as described in Section 2.1. The differences of overall *R*_free_ averaged over the free sets are negative up to 1.50 Å resolution [Fig. 2 ▸(*j*)]. CC* remains higher than CC_work_ in the whole resolution range for all the refined models. Moreover, the trend of *R*_gap_ [Fig. 2 ▸(*i*)] shows a moderate decrease for higher resolution going up to 1.42 Å when shells with a width of 0.01 Å were analyzed in the third run of paired refinement using the original free flag 0. To conclude, our calculations indicate that the data improve the model up to 1.50 Å resolution. This suggestion originates from the complete cross-validation protocol which should always be considered when deciding on the high-resolution cutoff.

### 3.4. Endo­thia­pepsin in complex with fragment B53   

In the cases reported above, the improvement of structure models using paired refinement was shown on statistical criteria. However, the increase in information gained from the data may also be shown by the interpretability of electron-density maps. Such enhancement was already reported for the crystal structure of the prokaryotic sodium channel pore (improvement from 4.0 to 3.5 Å resolution) and on the crystal structure of the YfbU protein from *E. coli* (improvement from 3.1 to 2.5 Å resolution) (Karplus & Diederichs, 2015 ▸). To demonstrate this effect using *PAIREF*, we reprocessed the diffraction data from the crystal structure of endothia­pepsin (EP) from *C. parasitica* in complex with fragment B53 (PDB entry 4y4g; Huschmann *et al.*, 2016 ▸) using *XDS*. The data set originates from a fragment screening project; fragment B53 has a partial occupancy.

**Figure 3:**
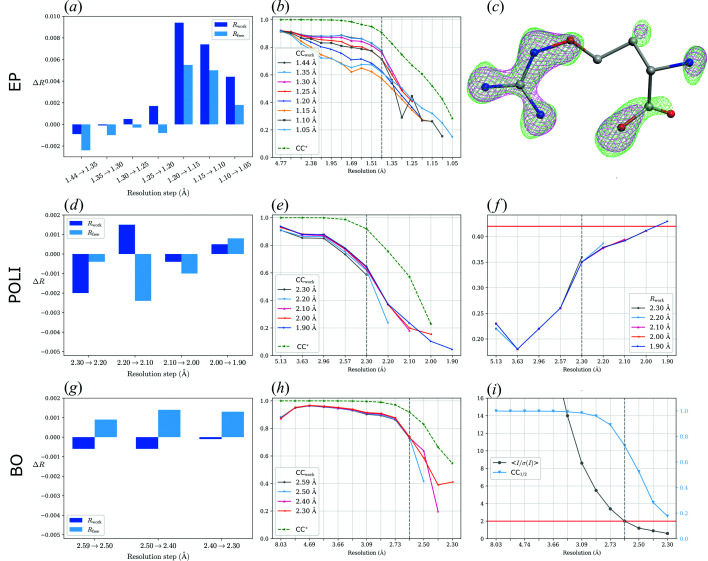
Results from paired refinement for EP (*a*)–(*c*), POLI (*d*)–(*f*) and BO (*g*)–(*i*). Note for bar charts showing the differences in the overall *R* values: for each incremental step of resolution for *X*→*Y*, the *R* values were calculated at resolution *X*. EP: (*a*) differences in the overall *R* values; resolution shells with a width of 0.05 Å were added stepwise. A systematic decrease in *R*_free_ was observed up to 1.20 Å. (*b*) CC* remains higher than CC_work_ in the whole resolution range for all the refined models. (*c*) Improvement in electron-density quality of the partially occupied fragment B53. Omit maps after refinement up to 1.44 (magenta) and 1.20 Å (green) are contoured at a level of 0.56 e Å^−3^. Atomic positions of the fragment molecule originate from PDB entry 4y4g (Huschmann *et al.*, 2016 ▸). The graphic was rendered in *CCP4mg* (McNicholas *et al.*, 2011 ▸). POLI: (*d*) differences in the overall *R* values; resolution shells with a width of 0.10 Å were added stepwise. (*e*) Comparison of CC* and CC_work_ of refined models. (*f*) *R*_work_ of refined models. The level *R*_work_ = 0.42 is shown as a red line. BO: (*g*) differences in the overall *R* values; resolution shells with a width of 0.10 Å were added stepwise. (*h*) Comparison of CC* and CC_work_ of refined models. (*i*) 〈*I*/σ(*I*)〉 and CC_1/2_ of the diffraction data depending on resolution; the level 〈*I*/σ(*I*)〉 = 2 is shown as a red line.

The data were originally processed up to 1.44 Å resolution with an 〈*I*/σ(*I*)〉 value of 2 in the highest resolution shell (1.52–1.44 Å). Here, we tried to simulate the regular workflow of model building and structure refinement. We removed all solvent molecules including ligands from the deposited model. The atomic coordinates were perturbed as done previously, the ADPs were manually set to their mean value of 16 Å^2^. Subsequently, 15 cycles of restrained refinement using anisotropic ADPs were performed with *REFMAC*5. These procedures were later followed by *PAIREF* calculations up to a resolution of 1.05 Å. According to our results, the optimal high-resolution limit was set to 1.20 Å [Fig. 3 ▸(*a*)] since positive *R*_free_ differences are observed for the higher resolution shells.

Inclusion of more intensities in the working data set considerably improved the quality of the omit map belonging to the partially occupied ligand [Fig. 3 ▸(*c*)]. In general, we expect that the greatest improvement in interpretability will occur for weak density features because the noise level of the map decreases due to improved phases resulting from a more accurate model. This will not significantly influence the observation of atoms with strong density. However, for a feature in the electron-density map that is close to the lower contour levels used in interpreting the map, having a bit less noise will have a higher impact on the reliability and interpretability of the electron-density map. In our case, this effect was observed in the stage of ligand and solvent building, which may be valuable especially in difficult cases and with low-occupied ligands.

### 3.5. Interferon gamma   

All the above-mentioned cases are high-resolution crystal structures. The crystal structure of interferon gamma from *P. olivaceus* (POLI) was previously determined at a medium resolution of 2.3 Å (Zahradník *et al.*, 2018 ▸). Moreover, the data exhibited severe anisotropy. Resolution limits were estimated in the range from 2.26 to 2.71 Å, according to the criterion of 〈*I*/σ(*I*)〉 being higher than 1.5 in the highest resolution shell (Evans & Murshudov, 2013 ▸). The data were reprocessed in *XDS* up to 1.9 Å resolution. The deposited structure (PDB entry 6f1e; Zahradník *et al.*, 2018 ▸) was refined using all of the reflections in the final refinement step. However, we used the last model refined using work reflections only in our paired refinement.

Several parameters were used to evaluate the high-resolution cutoff. Monitoring of *R*_free_ differences suggests a high-resolution cutoff at 2.0 Å [see Fig. 3 ▸(*d*)]. The value of *R*_work_ of the model refined at 1.9 Å calculated against the data in the highest resolution shell (2.0–1.9 Å) is high: 0.43 [Fig. 3(*f*) ▸], *i.e.* it exceeds the *R* value of a perfect model refined against random data (see Section 2.2). We suggest omitting the highest resolution shell in further refinement and cutting the data at 2.0 Å resolution. Poor CC* values in the high resolution are probably caused by the anisotropy of the diffraction data which affects the correlation between reflections. These results show that the decision on diffraction data resolution should not be based only on a single/certain value of data quality indicator, but on a more comprehensive evaluation of the available data.

### 3.6. Bilirubin oxidase   

The choice of the structure refinement program and parameters of refinement are the most decisive tools in paired refinement. *PAIREF* supports broad modification of structure refinement protocols using a command file for *REFMAC*5, including modification of ligand libraries. To demonstrate this functionality, we have analyzed the crystal structure of bili­rubin oxidase in complex with ferricyanide (BO) (PDB entry 6i3j). The structure was previously refined at 2.59 Å resolution with 〈*I*/σ(*I*)〉 equal to 2 in the highest resolution shell (Koval’ *et al.*, 2019 ▸) as shown in Fig. 3 ▸(*i*).

We have reprocessed the diffraction data up to a resolution of 2.3 Å with *XDS*. The last model originally refined using working reflections only was used as an input file for paired refinement. The library definitions for hexa­cyano­ferrate, weighting matrix and several external harmonic restraints were supplied to the refinement protocol (see the supporting information). In this case, no improvement in resolution can be expected according to *PAIREF*. Although the values of CC* are higher than CC_work_ and CC_free_ in the whole resolution range [Fig. 3 ▸(*h*)], an increase in *R*_free_ values indicates that the original high-resolution cutoff was set reasonably [Fig. 3 ▸(*g*)].

To further prove this, we ran the paired refinement protocol with 2.8 Å resolution as a starting resolution. At such low resolution, it was important to perform moderate atomic coordinate perturbation (mean shift 0.02 Å); the ADPs were set to their mean value of 35 Å^2^. In this case, paired refinement suggested the data should be cut at 2.6 Å resolution, which was the original conservative cutoff (see the supporting information).

In addition, we ran the paired refinement protocol starting at 2.59 Å resolution which was not supplied with the external harmonic restraints. An apparent improvement up to 2.5 Å resolution was observed in the data quality indicators. However, refinement lacking the important restraints led to unacceptable geometry of hexa­cyano­ferrate molecules and of several amino acid residues (away from the active site) in the output files and could not be accepted as a positive result. Analysis of the geometry of the refined model is beyond the scope of the *PAIREF* program as it is not implemented. Therefore, it remains the user’s responsibility to perform such analysis. To that end, *PAIREF* provides direct links to input, output and log files from all calculation procedures.

### 3.7. Impact of the model quality   

We performed a limited analysis of the impact of the starting model quality on results from paired refinement. We selected the EP and POLI data sets as examples of structures solved using molecular replacement and an experimental phasing method, respectively. Several models from different model building stages were used in the analysis.

#### 3.7.1. Molecular replacement and the EP data set   

**Figure 4:**
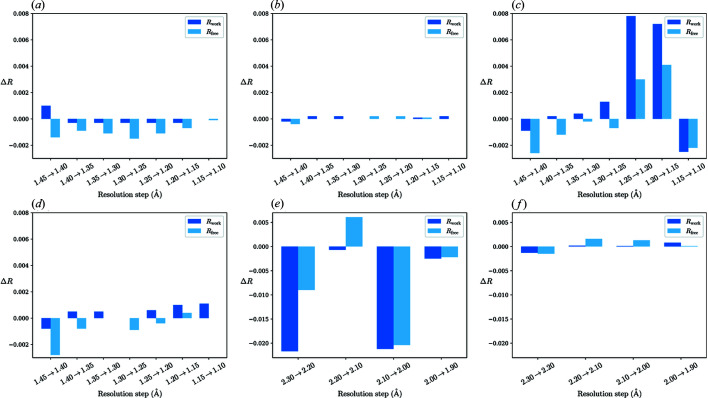
Paired refinement results for models from different building stages: EP (*a*)–(*d*) and POLI (*e*)–(*f*) data sets. For each incremental step of resolution for *X*→*Y*, the *R* values were calculated at resolution *X*. EP: resolution shells with a width of 0.05 Å. (*a*) Model after molecular replacement using a penicillopepsin structure. (*b*) Protein model as built by *ARP*/*wARP*. (*c*) Original model of endothia­pepsin without solvent molecules (PDB entry 4y4g). (*d*) Structure of endothia­pepsin as deposited in the PDB. (*e*)–(*f*) POLI: resolution shells with a width of 0.10 Å. (*e*) Poly-Ala model built by *SHELXE* into the experimental map. (*f*) Complete protein model without solvent molecules.

We solved the structure using the molecular replacement method with *Phaser* (McCoy *et al.*, 2007 ▸). The crystal structure of penicillopepsin (54% identity, 67% similarity; PBD entry 2wea; Ding *et al.*, 1998 ▸) was used as a search model. Subsequently, the protein chain was built automatically by *ARP*/*wARP* (Langer *et al.*, 2008 ▸) at the starting resolution (1.45 Å). Altogether, we analyzed four stages of the model building: (i) model placed by molecular replacement (*i.e.* containing the penicillopepsin sequence), (ii) the protein chain built by *ARP*/*wARP*, (iii) the original model of the final structure (PDB entry 4y4g) without solvent and (iv) the final complete deposited model [Figs. 4 ▸(*a*)–4(*d*)]. We used an identical setup for all the paired refinement protocols. Initially, the coordinates were perturbed by an average of 0.25 Å and the ADPs were set to their mean value, followed by 250 refinement cycles at the starting resolution (required for refinement convergence). Then, high-resolution shells with a width of 0.05 Å were added stepwise (see the supporting information).

Surprisingly, utilization of the data in the whole resolution range (up to 1.10 Å) is suggested when using a distant protein model correctly placed in the asymmetric unit. In contrast to this, improvement only up to 1.30 Å is observed using the model after complete protein rebuilding with *ARP*/*wARP*. Use of a protein model with no solvent molecules suggests the application of a high-resolution cutoff at 1.25 Å and for the most complete model at 1.20 Å.

#### 3.7.2. Experimental phasing and the POLI data set   

The crystal structure of interferon gamma from *P. olivaceus* was solved using SAD phasing. The following stages of model building were analysed: a poly-Ala model from *SHELXE* (Sheldrick, 2002 ▸), a complete protein model without solvent from *PHENIX AutoBuild* (Terwilliger *et al.*, 2008 ▸) [Figs. 4 ▸(*e*) and 4(*f*)] and the model prior to the final refinement [Fig. 3 ▸(*d*)] at the starting resolution (2.3 Å). Here we used optimized parameters of the paired refinement protocol for each specific model (see the supporting information).

The use of incomplete models in paired refinement suggested the application of a high-resolution cutoff of 2.2 Å, while the use of the most complete model a cutoff of 2.0 Å. Given both examples mentioned above, it can be stated that the model quality and completeness may play a significant role in the results from paired refinement.

## 4. Limitations and further development   

Amongst the hundreds of trials we performed, we did not register any failure of *PAIREF* itself. However, in a few cases, the external programs may fail to report an appropriate value, which may cause the crash of the *PAIREF* run. These cases were observed mostly at unreasonable resolution, *e.g.* the third or fourth resolution shell that should have already been omitted, or during analysis of very thin shells (*e.g.* 0.01 Å).

Results of paired refinement are strongly influenced by the structure refinement protocol (and in some cases also by the specific *REFMAC*5 version). In most of the cases mentioned above, a possible improvement in model accuracy owing to the use of higher-resolution data was detected using *PAIREF*. However, no improvement from the conservative cutoff was observed in the case of bilirubin oxidase.

The main focus of our further development will be the implementation of structure refinement using *phenix.refine*. Most of the procedures cannot be parallelized. Nevertheless, the parallelization of the complete cross-validation protocol is planned to significantly reduce computational time. Moreover, the inclusion of other monitoring statistics – *e.g. R*_complete_ (Luebben & Gruene, 2015 ▸) – in the final report is under development.

## 5. Discussion   

In macromolecular refinement, the maximum amount of valuable data should be used to obtain the best possible structural models. Hence, evaluation of data significance should be based on novel approaches. This involves the implementation of correlation coefficients and simultaneous monitoring of trends of several statistics that are directly linked to the quality of the refined model. Paired refinement is currently generally accepted as the optimal protocol for the determination of high-resolution cutoff. The *PAIREF* program is a command-line tool that performs such an analysis and creates a compact report for users to make a self-contained decision on the data limit.

In one of the examples documented here, we first analyzed the progress of the paired refinement procedure as well as the *PAIREF* functionality on data that have been artificially generated from a known structure. This structure later served as a target to monitor the convergence of the refined models. Continuous improvement in agreement between the original structure and models from paired refinement was observed in a range where our criteria suggested acceptance of further data. Here, the RMSD calculations showed that use of the high-resolution cutoff suggested by paired refinement produces models closest to the truth. The gap between CC_work_ and CC* visible for all projects except SIM corresponds to the *R*-value gap discussed by Holton *et al.* (2014 ▸), and is due to deficiencies in modelling the experiment.

We also tested the program on five other real cases, some of them previously used in paired refinement. In four cases, we showed that the model could be further improved by the use of data beyond conservative cutoffs. Our program is able to successfully reproduce two particular paired refinement protocols that were published previously [TL in the work by Winter *et al.* (2018 ▸) and CDO in the work by Karplus & Diederichs (2012 ▸)] and the results obtained are in good agreement with the original ones. Slight differences could be caused by the use of a newer version of *REFMAC*5 (in the case of TL), or by the utilization of other refinement software and the absence of an automatic solvent update during paired refinement (in the case of CDO).

In the case of bilirubin oxidase, an agreement in the high-resolution estimation between the conservative and paired refinement approach was observed. In all reported cases, the values of 〈*I*/σ(*I*)〉 and CC_1/2_ are in the ranges from 0.1 to 1.7 and from 0.027 to 0.524, respectively, all in the highest accepted resolution shell. Therefore, it is clear that a resolution cutoff based purely on certain values of these statistics does not correspond to the information content in the last or next additional resolution shell, as shown in previous works (Karplus & Diederichs, 2012 ▸, 2015 ▸; Diederichs & Karplus, 2013 ▸; Evans & Murshudov, 2013 ▸; Winter *et al.*, 2018 ▸).

The addition of high-resolution reflections suggested by the paired refinement results influences the amount of experimental data used in structure refinement as well as the overall agreement of the model to the data. In addition, it produces cleaner and more detailed maps which enable further manual improvement and removal of model errors by refinement. In the case of the data set from fragment screening (EP), we demonstrated that the involvement of valid data from higher resolution shells may have a positive impact on the quality of the electron-density map. Such an effect is clearly useful for low-occupancy ligands, partially disordered regions, alternative positions or low-resolution data.

We tested the influence of model quality on the results from paired refinement. We randomly chose a distant model for molecular replacement of the structure of endothia­pepsin and simulated the procedure of structure building and refinement. We also used three models from various stages of structure determination of interferon gamma from *Paralichthys olivaceus*. In these two cases, we observed that the use of a poor starting model suggested a lower high-resolution cutoff than the use of the most complete models. This notwithstanding, the use of a (partially) incorrect model may also result in a misleading suggestion, *e.g.* inclusion of the whole resolution range. Therefore, the input structure model should be selected carefully; paired refinement is particularly sensible in the final stage of structure refinement.

*PAIREF* worked well for the examples described using this general protocol: (i) processing of diffraction data at (almost) the full resolution; (ii) provisional resolution cutoff according to a conservative criterion, structure solution, model building and refinement; (iii) paired refinement with sufficient model quality at a later stage of model refinement.

With the introduction of paired refinement into X-ray crystallography, the high-resolution diffraction limit has gained a new meaning, as the only criterion for the data cutoff is now the ‘additional value’ of the data in model refinement. Following the current trends in diffraction data evaluation, resolution cannot be directly related to a specific value of the conventional indicators of diffraction data quality.

Reflections that were added during the paired refinement protocol generally represent data with the lowest information content. Since they come from the highest resolution shells, their 〈*I*/σ(*I*)〉 is lower, *R*_meas_ higher and CC_1/2_ lower. Nonetheless, they may represent a significant portion of the data. For most of the cases reported above, the reflections added through paired refinement account for more than 40% of all data. This of course is highly dependent on the conservative criteria that were used previously, before the paired refinement protocol was applied. Moreover, paired refinement has shown its importance for the improvement of structure models or even interpretability of electron-density maps.

## Supplementary Material

Supporting data. DOI: 10.1107/S2052252520005916/mf5044sup1.pdf

Paired refinement under control of PAIREF - examples: http://doi.org/10.5281/zenodo.3687267

## Figures and Tables

**Table 1:** Data collection and merging statistics Values for the highest resolution shell in the case of conservative cutoff are given in parentheses () and for the cutoff chosen as optimal are given in square brackets []. SIM represents a simulated data set generated by *MLFSOM* (Holton *et al.*, 2014 ▸).

Data set	SIM	TL	CDO	EP	POLI	BO†
Data set DOI	10.15785/SBGRID/746	10.5281/zenodo.49559	10.15785/SBGRID/751	10.18430/m34y4g	10.5281/zenodo.3369718	10.18430/m36i3j
X-ray source	*MLFSOM*	BL I03, Diamond Light Source	BL 5.0.1. Advanced Light Source	BL14.1, BESSY II	BL14.1, BESSY II	BL14.1, BESSY II
Wavelength (Å)	1.0000	1.2276	0.9774	0.9184	0.9184	0.9184
Detector	Simulated PILATUS 6M	PILATUS 6M	ADSC	PILATUS 6M	PILATUS 6M	MAR mosaic CCD
Temperature (K)	N/A	N/A	100	100	100	100
Crystal-to-detector distance (mm)	150.0	209.4	150.0	180.8	446.3	313.5
Oscillation angle/range (°)	1/100	0.1/720	1/218	0.1/200	0.1/360	0.5/108.5
Resolution range (Å)	38.64–1.30 (1.98–1.72) [1.40–1.30]	79.98–1.50 (1.90–1.80) [1.60–1.50]	41.96–1.50 (2.10–2.00) [1.60–1.50]	49.64–1.20 (1.51–1.44) [1.25–1.20]	47.32–2.00 (2.38–2.30) [2.10–2.00]	47.35–2.59 (2.67–2.59) 〈2.59–2.50〉†
Space group	*P*4_3_2_1_2	*P*6_1_22	*P*4_3_2_1_2	*P*2_1_	*P*2_1_2_1_2_1_	*F*222
Unit-cell parameters						
*a* (Å)	77.24	92.35	57.63	45.20	58.27	134.5
*b* (Å)	77.24	92.35	57.63	73.10	79.76	204.1
*c* (Å)	38.66	127.71	122.39	52.57	94.64	227.0
α (°)	90.00	90.00	90.00	90.00	90.00	90.00
β (°)	90.00	90.00	90.00	109.25	90.00	90.00
γ (°)	90.00	120.00	90.00	90.00	90.00	90.00
Wilson *B* factor (Å^2^)	12.2	20.3	24.2	15.5	64.5	44.5
No. reflections	166742 (30516) [16791]	3714005 (341691) [510558]	522379 (33610) [60331]	371954 (29343) [42646]	393534 (23854) [49772]	399548 (27236) 〈27420〉†
No. unique reflections	28932 (4336) [5345]	50760 (4391) [8252]	33898 (1938) [5862]	97408 (7460) [10944]	30377 (1928) [4021]	48468 (4177) 〈5353〉†
No. additional unique reflections‡	16029 {1.72–1.30}	20518 {1.80–1.50}	25117 {2.00–1.50}	40250 {1.44–1.20}	10202 {2.30–2.00}	0
Multiplicity	5.8 (7.0) [3.1]	73.2 (77.8) [61.9]	15.4 (17.3) [10.3]	3.8 (3.9) [3.9]	13.0 (12.4) [12.4]	8.2 (6.5) 〈5.1〉†
Completeness (%)	98.6 (99.9) [93.4]	97.6 (98.3) [91.8]	100.0 (100.0) [100.0]	96.8 (96.3) [94.6]	99.7 (100.0) [98.5]	100.0 (100.0) 〈99.8〉†
Mean *I*/σ(*I*)	5.9 (4.0) [0.3]	13.3 (4.4) [0.8]	22.7 (18.1) [0.9]	6.6 (1.7) [0.5]	9.0 (0.9) [0.1]	13.8 (1.7) 〈1.2〉†
*R*_meas_	0.131 (0.254) [2.233]	0.223 (1.143) [4.828]	0.150 (0.334) [2.133]	0.117 (0.777) [2.500]	0.154 (2.907) [17.721]	0.150 (1.143) 〈1.338〉†
*R*_pim_	0.052 (0.094) [1.153]	0.025 (0.127) [0.598]	0.037 (0.079) [0.654]	0.059 (0.385) [1.247]	0.043 (0.816) [4.963]	0.052 (0.445) 〈0.584〉†
CC_1/2_	0.992 (0.971) [0.179]	1.000 (0.961) [0.445]	0.999 (0.996) [0.437]	0.998 (0.694) [0.225]	0.999 (0.578) [0.027]	0.997 (0.652) 〈0.524〉†
Resolution range (Å)§	38.64–1.35	79.98–1.43	41.96–1.42	49.64–1.11	47.32–1.90	47.35–2.30
CC*	0.998 (0.993) [0.551]	1.000 (0.990) [0.785]	1.000 (0.999) [0.780]	0.999 (0.905) [0.606]	1.000 (0.856) [0.229]	0.999 (0.888) 〈0.829〉†

† For the BO data set, values for a resolution shell beyond the optimal cutoff are listed in angled brackets 〈〉.

‡ Number of additional reflections suggested by paired refinement results to be involved in the refinement in contrast to the starting resolution. Added resolution range, in Å, is given in {} brackets.

§ Range where CC_1/2_ is significantly different from 0 at the 1:1000 level.

**Table 2:** Structure refinement and validation statistics Values are listed for the models refined at the starting and the optimal resolution in square brackets []. *ΔR* is the difference between *R* values relating to the model refined at the optimal and the starting resolution (both calculated at the starting resolution). SIM is a simulated data set generated by *MLFSOM* (Holton *et al.*, 2014 ▸).

Data set	SIM	TL	CDO†	EP	POLI	BO‡
Resolution range (Å)	38.64–1.72 [38.64–1.30]	79.98–1.80 [79.98–1.50]	41.96–2.00 [41.96–1.50]	49.64–1.44 [49.64–1.20]	47.32–2.30 [47.32–2.00]	47.35–2.59 〈47.35–2.50〉‡
Optical resolution (Å)	1.41 [1.25]	1.52 [1.42]	1.50 [1.30]	1.30 [1.15]	2.16 [2.08]	2.01 〈1.99〉‡
*R*_work_	0.0605 [0.1047]	0.1580 [0.1742]	0.1560 (σ = 0.0010) [0.2070 (σ = 0.0010)]	0.2017 [0.2241]	0.2236 [0.2412]	0.1754 〈0.1881〉‡
Δ*R*_work_	−0.0011	0.0028	0.0048	0.0026	−0.0003	0.0002
*R*_free_	0.0711 [0.1112]	0.1954 [0.2037]	0.2060 (σ = 0.0080) [0.2380 (σ = 0.0070)]	0.2566 [0.2656]	0.2972 [0.3152]	0.2408 〈0.2498〉‡
Δ*R*_free_	−0.0042	−0.0023	−0.0090	−0.0051	−0.0016	0.0003
CC_work_	0.9822 [0.9826]	0.9615 [0.9630]	0.9590 (σ = 0.0020) [0.9650 (σ = 0.0010)]	0.9436 [0.9306]	0.9199 [0.9387]	0.9450 〈0.9471〉‡
CC_free_	0.9915 [0.9920]	0.9467 [0.9498]	0.9400 (σ = 0.0200) [0.9500 (σ = 0.0100)]	0.9177 [0.9069]	0.8678 [0.8690]	0.9151 〈0.9168〉‡
Average ADP (Å^2^)	13.67 [13.59]	22.55 [23.43]	14.47 [19.17]	13.10 [12.76]	70.09 [68.17]	45.10 〈46.87〉‡
RMSD bond lengths (Å)	0.012 [0.013]	0.012 [0.012]	0.011 [0.013]	0.017 [0.014]	0.012 [0.013]	0.008 〈0.008〉‡
RMSD bond angles (°)	1.915 [1.942]	1.649 [1.707]	1.739 [1.853]	1.846 [1.797]	1.829 [2.005]	1.326 〈1.654〉‡
No. of non-hydrogen atoms	1217	2816	1836	2459	2286	9511
Ramachandran: favoured (%)	91.3 [92.1]	93.6 [96.6]	97.3 [97.3]	97.4 [97.0]	93.1 [94.2]	90.8 〈90.7〉‡
Ramachandran: outliers (%)	0.0 [0.0]	1.0 [1.0]	0.0 [0.0]	0.0 [0.3]	1.5 [1.5]	1.4 〈1.4〉‡

† In the case of complete cross-validation (data set CDO), *R* values and CC values averaged over all 20 free reflection sets and the associated standard deviation σ are listed. The remaining statistics relate to the refinements with free reflection set 0.

‡ For the BO data set, values for a resolution shell beyond the optimal cutoff are listed in angled brackets 〈〉.
