# Supplementary material for: Paired refinement under the control of PAIREF
Source: IUCrJ. 2020 Jun 10;7(Pt 4):681–92. doi: 10.1107/S2052252520005916 (PMC7340264; doi:10.1107/S2052252520005916)
Supplement: Supplementary file 1 [file m-07-00681-sup1.pdf]

IUCrJ

Volume 7 (2020)

Supporting information for article:

Paired refinement under the control of *PAIREF*

Martin Malý, Kay Diederichs, Jan Dohnálek and Petr Kolenko

All the refinement runs presented in this paper were performed with *REFMAC*5 version 5.8.258.

Installation of *PAIREF*:
PAIREF depends on the *CCP*4 package. Run a following command in Unix shell (GNU/Linux, MacOS) or in *CCP4CONSOLE* (MS Windows):

```
cctbx.python -m pip install pairef --user --no-deps
```

S1 Simulated data set of lysozyme (SIM)

Perturbation of the reference structure model:

```
phenix.pdbtools set_b_iso=15 shake=0.25 1H87_single.pdb
file_name=1H87_single_shaken.pdb
```

Run with a resolution shell width of 0.10 Å (Fig. 2a-c):

```
cctbx.python -m pairef --XYZIN 1H87_single_shaken.pdb --HKLIN 1H87_dui_R.mtz -u
1H87_dui_unmerged.mtz -i 1.72 -r 1.6,1.5,1.4,1.3,1.2 --prerefinement-ncyc 13 --ncyc 6
-p 1H87_dui
```

```
# Shell (A) Rwork(init) Rwork(fin) Rwork(diff) Rfree(init) Rfree(fin) Rfree(diff)
1.72A->1.60A    0.0609      0.0592    -0.0017      0.0712     0.0681    -0.0031
1.60A->1.50A    0.0655      0.0653    -0.0002      0.0746     0.0734    -0.0012
1.50A->1.40A    0.0733      0.0733     0.0000      0.0812     0.0805    -0.0007
1.40A->1.30A    0.0848      0.0847    -0.0001      0.0938     0.0935    -0.0003
1.30A->1.20A    0.1047      0.1049     0.0002      0.1112     0.1113     0.0001
```

RMS coordinates differences were calculated in PyMOL using function `rms_cur()`.
RMS of ADP differences were calculated according to formula

$$RMSD_{\mathrm{ADP}} = \sqrt{\frac{\sum_{i=1}^{N}[B_{1,i} - \overline{B_1} - B_{2,i} + \overline{B_2}]^2}{N}},$$

where both structure models consist of N atoms with ADPs $B_{1,1}$, $B_{1,2}$, …, $B_{1,N}$, and $B_{2,1}$, $B_{2,2}$, …, $B_{2,N}$. Bars denote average values.

Merging statistics in resolution bins:

```
#shell d_max  d_min   #obs  #uniq   mult.  %comp      <I>  <I/sI>   r_mrg  r_meas   r_pim  cc1/2  cc_ano     cc*
01     38.62   3.43  11225   1756    6.39  99.83    725.8    27.2   0.060   0.066   0.027  0.985   0.207  0.9962
02      3.43   2.43  20639   2965    6.96  99.83    264.6    18.8   0.059   0.064   0.024  0.997   0.618  0.9992
03      2.43   1.98  25070   3846    6.52 100.00    108.4     9.5   0.105   0.114   0.043  0.988   0.404  0.9970
04      1.98   1.72  30516   4336    7.04  99.93     40.8     4.0   0.235   0.254   0.094  0.971   0.156  0.9926
05      1.72   1.60  19736   3054    6.46 100.00     20.7     2.0   0.431   0.468   0.180  0.901   0.080  0.9736
06      1.60   1.50  19671   3305    5.95  99.70     13.1     1.1   0.731   0.800   0.317  0.752   0.008  0.9265
07      1.50   1.40  23094   4325    5.34  99.61      8.9     0.6   1.110   1.230   0.515  0.513   0.024  0.8235
08      1.40   1.30  16791   5345    3.14  93.44      5.3     0.3   1.890   2.233   1.153  0.179   0.021  0.5510
09      1.30   1.20   3690   2566    1.44  33.03      2.7     0.1   3.379   4.476   2.900  0.034  -0.384  0.2564
```

S2 Thermolysin (TL)

Run with a resolution shell width of 0.10 Å (Fig. 2d-e):

```
cctbx.python -m pairef --XYZIN 3n21_edit05_refmac1.pdb --HKLIN
AUTOMATIC_DEFAULT_free_R.mtz -u AUTOMATIC_DEFAULT_scaled_unmerged.mtz -i 1.80 -n 5 -s
0.10 -p TL_step0-10A
```

Run with a resolution shell width of 0.01 Å (Fig. 2f):

```
cctbx.python -m pairef --XYZIN 3n21_edit05_refmac1.pdb --HKLIN
AUTOMATIC_DEFAULT_free_R.mtz -u AUTOMATIC_DEFAULT_scaled_unmerged.mtz -i 1.80 -n 49 -s
0.01 -p TL_step0-01A
```

Merging statistics in resolution bins:

#shell	d_max	d_min	#obs	#uniq	mult.	%comp	<I>	<I/sI>	r_mrg	r_meas	r_pim	cc1/2	cc_ano	cc*
01	79.99	4.77	125965	1832	68.76	100.00	26.5	56.8	0.071	0.072	0.008	1.000	0.700	1.0000
02	4.77	3.37	232091	3108	74.68	99.97	28.4	56.4	0.088	0.089	0.010	1.000	0.503	1.0000
03	3.37	2.75	301747	3938	76.62	99.92	11.7	33.1	0.150	0.151	0.017	0.999	0.355	0.9997
04	2.75	2.38	342932	4589	74.73	99.59	5.0	19.5	0.255	0.257	0.029	0.997	0.231	0.9992
05	2.38	2.13	396759	5102	77.77	99.20	2.8	13.4	0.379	0.381	0.042	0.994	0.147	0.9985
06	2.13	1.94	427018	5775	73.94	98.79	1.5	8.4	0.595	0.599	0.068	0.986	0.049	0.9965
07	1.94	1.80	455635	5900	77.23	98.38	0.6	4.8	1.040	1.046	0.117	0.965	0.052	0.9911
08	1.80	1.70	403806	5435	74.30	97.96	0.3	2.8	1.741	1.753	0.200	0.723	0.010	0.9161
09	1.70	1.60	517662	6831	75.78	97.47	0.2	1.6	2.872	2.891	0.326	0.657	0.022	0.8905
10	1.60	1.50	510558	8252	61.87	91.80	0.1	0.8	4.789	4.828	0.598	0.445	0.009	0.7848
11	1.50	1.40	299987	7139	42.02	61.05	0.0	0.2	9.608	9.727	1.445	0.077	0.023	0.3781
12	1.40	1.31	84121	4154	20.25	32.19	0.0	-0.1	20.319	20.855	4.431	0.019	0.010	0.1931

S3 Cysteine dioxygenase (CDO)

Run with a resolution shell width of 0.10 Å - free flag 0 (Fig. 2g-h):

```
cctbx.python -m pairef --HKLIN CDO_R.mtz --XYZIN 2B5H_edit_refmac1.pdb -u XDS_ASCII.HKL -i 2 -r 1.9,1.8,1.7,1.6,1.5,1.42 -p CDO_step0-10A
```

Run with a resolution shell width of 0.01 Å - free flag 0 (Fig. 2i):

```
cctbx.python -m pairef --HKLIN CDO_R.mtz --XYZIN 2B5H_edit_refmac1.pdb -u XDS_ASCII.HKL -i 2 -s 0.01 -n 58 -p CDO_step0-01A
```

Run with a resolution shell width of 0.10 Å - complete cross-validation (Fig. 2j-l):

```
cctbx.python -m pairef --HKLIN CDO_R.mtz --XYZIN 2B5H_edit_refmac1.pdb -u XDS_ASCII.HKL -i 2 -r 1.9,1.8,1.7,1.6,1.5,1.42 --complete --prerefinement-ncyc 20  --prerefinement-shake-sites 0.25 --prerefinement-reset-bfactor -p CDO_step0-10A_complete
```

Merging statistics in resolution bins:

#shell	d_max	d_min	#obs	#uniq	mult.	%comp	<I>	<I/sI>	r_mrg	r_meas	r_pim	cc1/2	cc_ano	cc*
01	41.96	3.98	30502	2013	15.15	100.00	509.9	92.4	0.029	0.030	0.008	1.000	0.444	1.0000
02	3.98	2.83	55734	3335	16.71	100.00	382.3	74.0	0.048	0.049	0.012	1.000	0.178	1.0000
03	2.83	2.31	73318	4261	17.21	100.00	152.2	40.9	0.127	0.131	0.031	0.999	0.046	0.9997
04	2.31	2.00	86730	5014	17.30	100.00	100.2	23.4	0.254	0.261	0.062	0.997	0.009	0.9992
05	2.00	1.90	40614	2360	17.21	100.00	60.5	13.0	0.412	0.425	0.101	0.993	-0.011	0.9982
06	1.90	1.80	48612	2886	16.84	100.00	31.9	6.8	0.577	0.595	0.144	0.976	0.015	0.9939
07	1.80	1.70	60936	3619	16.84	100.00	16.1	3.5	0.854	0.881	0.214	0.933	0.108	0.9825
08	1.70	1.60	65470	4548	14.40	100.00	8.9	1.9	1.251	1.297	0.340	0.783	0.144	0.9372
09	1.60	1.50	60309	5862	10.29	100.00	4.9	0.9	2.023	2.130	0.653	0.441	-0.021	0.7824
10	1.50	1.42	27549	5842	4.72	98.83	2.9	0.3	3.175	3.557	1.527	0.074	0.004	0.3712

S4 Endothiapepsin in complex with fragment B53 (EP)

Perturbation of the structure model:

```
phenix.pdbtools set_b_iso=16 shake=0.25 4Y4G_noW_noLIG.pdb file_name=4Y4G_noW_noLIG_shaken.pdb
```

Run with a resolution shell width of 0.05 Å (Fig. 3a-c):

```
cctbx.python -m pairef --XYZIN 4Y4G_noW_noLIG_shaken.pdb --HKLIN EP_R.mtz -u XDS_ASCII.HKL -i 1.44 -r 1.35,1.30,1.25,1.20,1.15,1.10,1.05 --prerefinement-ncyc 15 -p EP
```

Merging statistics in resolution bins:

#shell	d_max	d_min	#obs	#uniq	mult.	%comp	<I>	<I/sI>	r_mrg	r_meas	r_pim	cc1/2	cc_ano	cc*
01	42.68	4.77	6511	1646	3.96	99.58	198.1	41.3	0.024	0.027	0.013	0.999	-0.001	0.9997
02	4.77	3.37	10618	2984	3.56	99.60	269.3	36.7	0.025	0.030	0.015	0.999	-0.098	0.9997
03	3.37	2.75	14924	3814	3.91	99.17	112.3	26.3	0.040	0.046	0.023	0.998	-0.031	0.9995
04	2.75	2.38	17054	4492	3.80	98.99	55.7	17.9	0.059	0.068	0.034	0.996	0.016	0.9990
05	2.38	2.14	17117	4812	3.56	98.61	41.7	13.8	0.074	0.087	0.045	0.993	0.031	0.9982
06	2.14	1.95	21801	5624	3.88	98.36	29.6	11.5	0.094	0.110	0.055	0.990	0.013	0.9975
07	1.95	1.80	24698	6237	3.96	98.10	15.4	7.3	0.156	0.180	0.089	0.976	0.013	0.9939
08	1.80	1.69	23067	6065	3.80	97.78	8.6	4.5	0.245	0.285	0.144	0.941	0.044	0.9847
09	1.69	1.59	25972	7046	3.69	97.40	5.5	3.0	0.361	0.423	0.217	0.871	0.043	0.9649
10	1.59	1.51	27217	6983	3.90	96.78	3.9	2.3	0.490	0.568	0.284	0.822	0.041	0.9499
11	1.51	1.44	29343	7460	3.93	96.32	2.8	1.7	0.672	0.777	0.385	0.694	0.035	0.9052
12	1.44	1.35	44398	11896	3.73	96.04	1.9	1.1	0.967	1.128	0.573	0.521	0.020	0.8277
13	1.35	1.30	30329	8072	3.76	95.49	1.3	0.7	1.359	1.585	0.806	0.385	0.032	0.7456
14	1.30	1.25	36280	9338	3.89	95.05	1.0	0.6	1.728	2.003	1.002	0.288	0.032	0.6687
15	1.25	1.20	42646	10944	3.90	94.61	0.8	0.5	2.158	2.500	1.247	0.225	0.038	0.6061
16	1.20	1.15	47423	12864	3.69	94.05	0.6	0.3	2.764	3.235	1.656	0.156	0.038	0.5195
17	1.15	1.10	58651	15099	3.88	93.26	0.5	0.2	3.569	4.138	2.070	0.099	0.037	0.4245
18	1.10	1.05	54538	15315	3.56	79.14	0.2	0.1	5.979	6.987	3.564	0.042	0.025	0.2839

S5 Interferon gamma (POLI)

Run with a resolution shell width of 0.1 Å (Fig. 3d-f):

```
cctbx.python -m pairef --XYZIN poli67_edit12_refmac1.pdb --HKLIN poli67_R.mtz -u
XDS_ASCII.HKL -i 2.3 -s 0.1 -n 4 --ncyc 10 --prerefinement-ncyc 10 -w 0.06 -p poli67
```

Merging statistics in resolution bins:

#shell	d_max	d_min	#obs	#uniq	mult.	%comp	<I>	<I/sI>	r_mrg	r_meas	r_pim	cc1/2	cc_ano	cc*
01	47.32	5.13	22369	1949	11.48	99.44	494.0	38.8	0.042	0.044	0.013	0.999	-0.214	0.9997
02	5.13	3.63	42232	3343	12.63	99.79	203.3	31.7	0.064	0.067	0.019	0.999	-0.163	0.9997
03	3.63	2.97	57138	4246	13.46	99.95	56.9	14.3	0.151	0.157	0.042	0.997	-0.166	0.9992
04	2.97	2.57	65007	5014	12.97	99.70	11.3	4.0	0.622	0.648	0.178	0.951	-0.025	0.9874
05	2.57	2.30	73887	5623	13.14	99.89	3.7	1.3	1.839	1.914	0.523	0.730	-0.014	0.9187
06	2.30	2.20	37405	2818	13.27	99.93	1.6	0.6	4.462	4.641	1.263	0.400	0.033	0.7559
07	2.20	2.10	45724	3363	13.60	99.91	0.9	0.3	7.623	7.920	2.129	0.196	-0.008	0.5725
08	2.10	2.00	49772	4021	12.38	98.51	0.3	0.1	16.989	17.721	4.963	0.027	0.008	0.2293
09	2.00	1.90	35920	4046	8.88	81.18	0.1	0.0	41.435	43.993	14.417	-0.132	-0.016	N/A

S6 Bilirubin oxidase (BO)

Run with a resolution shell width of 0.1 Å (Fig. 3g-h):

```
cctbx.python -m pairef --XYZIN BO_edit94_refmac1.pdb --HKLIN BO_R.mtz -u XDS_ASCII.HKL
--LIBIN merge_TRP-HIS_FC6.cif -c setting.com -r 2.5,2.4,2.3 -w 0.048 --prerefinement-
ncyc 10 -p BO
```

The file setting.com includes:

```
#  Hexacyanoferrate ligand definition in the working file.
# Residue numbers correspond to A613 to A616 and B611 to B615 in structure with PDB ID 6I3J, i.e. before
# renumbering in deposition
external harmonic residues from 801 A to 804 A sigma 0.03
external harmonic residues from 801 B to 805 B sigma 0.03
# protein residues
external harmonic residues from 139 B to 139 B sigma 0.01
external harmonic residues from 274 B to 274 B sigma 0.01
exte dist first chain A resi 463 atom CD second chain A resi 463 atom OE1 value 1.25 sigma 0.02
exte dist first chain B resi 492 atom CD second chain B resi 492 atom OE1 value 1.25 sigma 0.02
exte dist first chain A resi 503 atom CD second chain A resi 503 atom OE1 value 1.25 sigma 0.02
exte dist first chain B resi 143 atom CD second chain B resi 143 atom OE2 value 1.25 sigma 0.02
```

Run with a resolution shell width of 0.1 Å (2.8 Å resolution as a starting resolution):

```
cctbx.python -m pairef --XYZIN BO_edit94_refmac1.pdb --HKLIN BO_R.mtz -u XDS_ASCII.HKL
--LIBIN merge_TRP-HIS_FC6.cif -c setting.com -i 2.8 -r 2.7,2.6,2.5,2.4,2.3 -w 0.048 --
prerefinement-ncyc 10 --prerefinement-shake-sites 0.02 --prerefinement-reset-bfactor -
p BO_from2-80A
```

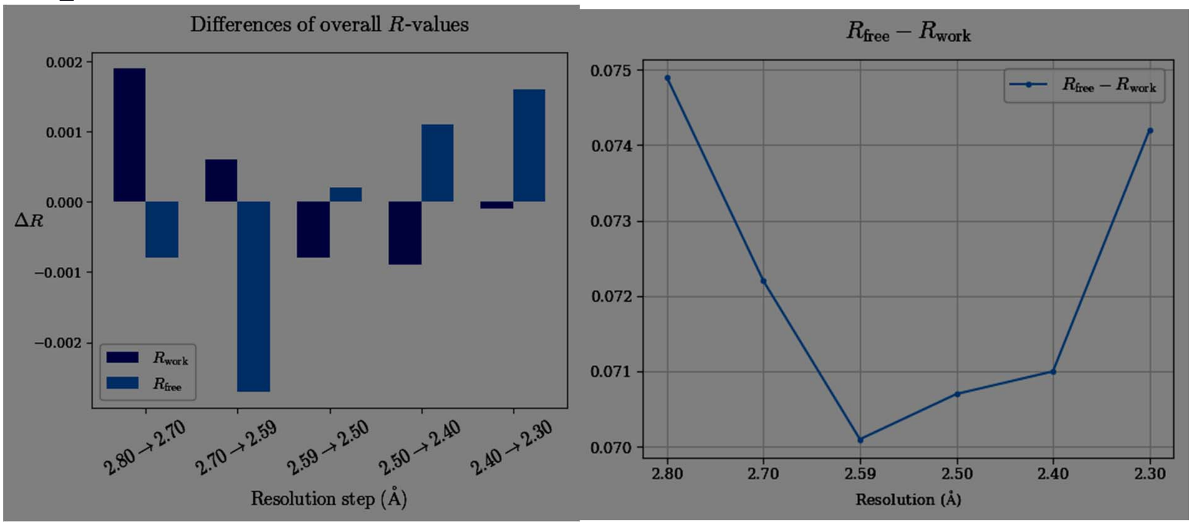

Merging statistics in resolution bins:

#shell	d_max	d_min	#obs	#uniq	mult.	%comp	<I>	<I/sI>	r_mrg	r_meas	r_pim	cc1/2	cc_ano	cc*
01	47.35	8.03	13105	1720	7.62	99.59	384.0	40.9	0.038	0.040	0.014	0.999	0.214	0.9997
02	8.03	5.73	23998	2921	8.22	100.00	193.6	28.5	0.063	0.067	0.023	0.998	0.092	0.9995
03	5.73	4.74	28851	3458	8.34	100.00	242.6	29.1	0.061	0.065	0.022	0.998	0.034	0.9995
04	4.74	4.10	36579	4349	8.41	100.00	258.3	27.6	0.065	0.070	0.024	0.998	0.016	0.9995
05	4.10	3.66	41914	4954	8.46	100.00	193.4	21.0	0.090	0.096	0.033	0.997	-0.002	0.9992
06	3.66	3.34	45996	5418	8.49	99.98	121.4	14.0	0.145	0.154	0.052	0.993	0.019	0.9982
07	3.34	3.09	50156	5898	8.50	100.00	73.1	8.6	0.247	0.263	0.089	0.984	0.017	0.9960
08	3.09	2.89	53641	6305	8.51	100.00	44.5	5.5	0.396	0.421	0.143	0.960	0.011	0.9897
09	2.89	2.73	54665	6426	8.51	100.00	27.6	3.4	0.650	0.691	0.235	0.891	0.016	0.9708
10	2.73	2.59	50143	7020	7.14	100.00	18.1	2.0	0.942	1.017	0.377	0.728	-0.006	0.9179
11	2.59	2.50	27420	5353	5.12	99.79	13.5	1.2	1.198	1.338	0.584	0.524	0.025	0.8293
12	2.50	2.40	27592	6777	4.07	97.54	11.2	0.9	1.396	1.613	0.785	0.283	0.008	0.6642
13	2.40	2.30	24246	7300	3.32	89.13	8.7	0.6	1.795	2.145	1.140	0.176	0.007	0.5471

S7 Impact of the model quality

S7.1 EP data set

Run with model after molecular replacement using a penicillopepsin structure (Fig 4a):

```
cctbx.python -m pairef --XYZIN EP_afterMR_2WEA.pdb --HKLIN EP_R.mtz -u XDS_ASCII.HKL -
i 1.45 -n 7 -s 0.05 --ncyc 10 --prerefinement-ncyc 250 --prerefinement-reset-bfactor -
-prerefinement-shake-sites 0.25 -p EP_afterMR_2WEA
```

Run with protein model as built by *ARP/wARP* (Fig 4b):

```
cctbx.python -m pairef --XYZIN EP_protein_chain_ARPwARP.pdb --HKLIN EP_R.mtz -u
XDS_ASCII.HKL -i 1.45 -n 7 -s 0.05 --ncyc 10 --prerefinement-ncyc 250 --prerefinement-
reset-bfactor --prerefinement-shake-sites 0.25 -p EP_protein_chain_ARPwARP
```

Run with original model of endothiapepsin without solvent molecules (Fig 4c):

```
cctbx.python -m pairef --XYZIN 4Y4G_noW_noLIG.pdb --HKLIN EP_R.mtz -u XDS_ASCII.HKL -i
1.45 -n 7 -s 0.05 --ncyc 10 --prerefinement-ncyc 250 --prerefinement-reset-bfactor --
prerefinement-shake-sites 0.25 -p EP_noW_noLIG
```

Run with deposited structure of endothiapepsin - PDB id 4Y4G (Fig 4d):

```
cctbx.python -m pairef --XYZIN 4Y4G.pdb --HKLIN EP_R.mtz -u XDS_ASCII.HKL -i 1.45 -n 7
-s 0.05 --ncyc 10 --prerefinement-ncyc 250 --prerefinement-reset-bfactor --
prerefinement-shake-sites 0.25 -p EP_4Y4G
```

S7.2 POLI data set

Run with poly-Ala model as built by *SHELXE* (Fig 4e):

```
Poly-Ala
cctbx.python -m pairef --XYZIN poli67-poliAla.pdb --HKLIN poli67_R.mtz -u
XDS_ASCII.HKL -i 2.3 -s 0.1 -n 4 --ncyc 10 --prerefinement-ncyc 55 --ncyc 60 -p
poli67-poliAla
```

Run with protein model without solvent molecules (Fig 4f):

```
No waters
cctbx.python -m pairef --XYZIN poli67-noW.pdb --HKLIN poli67_R.mtz -u XDS_ASCII.HKL -i
2.3 -s 0.1 -n 4 --ncyc 10 --prerefinement-ncyc 10 --ncyc 10 -w 0.06 -p poli67-nowaters
```
